# Beyond 20/20: How Laser Eye Surgery Boosts Quality of Life (SMILE vs. FS‐LASIK)

**DOI:** 10.1155/joph/7225757

**Published:** 2026-06-08

**Authors:** Pieter Juanarta, Susanti Natalya Sirait, R. Angga Kartiwa, Feti Karfiati, Rova Virgana, Budiman

**Affiliations:** ^1^ Universitas Padjadjaran Facultas Kedokteran, Jawa Barat, Indonesia; ^2^ Pusat Mata Nasional Rumah Sakit Mata Cicendo Bandung, Bandung, Indonesia

**Keywords:** FS-LASIK, patients’ quality of life, SMILE

## Abstract

**Purpose:**

Uncorrected refractive errors are the main cause of visual impairment globally and can be corrected with refractive surgeries, such as femtosecond laser–assisted in situ keratomileusis (FS‐LASIK) and small‐incision lenticule extraction (SMILE). The success rate of the procedure is determined by its efficacy, stability, predictability, and safety. Although these parameters are important, they should be accompanied by the patient’s subjective report to assess the success of the surgery. Research has shown that SMILE patients have a better quality of life owing to lower rates of postoperative symptoms and dry eyes. This study aimed to compare improvement in patient quality of life after the SMILE and FS‐LASIK procedures.

**Methods:**

This was an analytical observational study with a prospective design conducted at the PMN Cicendo Eye Hospital. Fifty‐seven patients met the inclusion criteria: 33 and 24 patients underwent the FS‐LASIK and SMILE procedures, respectively. Samples were collected using a purposive method at a LASIK center. Patients were asked to complete the NEI‐RQL 42 questionnaire, and an IDRA ocular surface analyzer examination was carried out at the Aesthetic and Dry Eye Clinic.

**Results:**

Patient characteristics were dominated by male sex, secondary education levels, and students. Significant differences in characteristics were found with greater refractive error in the FS‐LASIK group. A comparison of questionnaire score improvements revealed higher scores in the SMILE group, especially in terms of symptoms and average questionnaire scores.

**Conclusion:**

There was a better improvement in quality of life in patients after the SMILE procedure than after the FS‐LASIK procedure.

## 1. Introduction

Uncorrected refractive errors are the main cause of visual impairment globally, contributing to 20.9% of blindness cases and 52.9% of moderate and severe visual impairment cases. Research by Aldiana et al. showed that the rates of refractive errors in children in Bandung were 15.9%, and it was 12.1% for those without correction. With the development of technology in the field of ophthalmology, refractive surgery has become one of the main choices for patients with refractive errors to avoid the use of assistive devices. Several surgical techniques can be performed, such as LASIK and FS‐SMILE. LASIK is the most frequently performed corneal refractive surgery. The technology used to create LASIK flaps has evolved from microkeratomes to femtosecond lasers. Another refractive surgery that is often performed is the small‐incision lenticule extraction (SMILE) procedure, which corrects refractive errors using a femtosecond laser. With the SMILE procedure, complications associated with LASIK flap creation can be minimized, and there is less corneal nerve damage so that corneal sensitivity and biomechanics can be maintained [1–10].

Several studies have shown that patients who undergo SMILE have a better quality of life than those who undergo other refractive surgical procedures. Quality of life can be influenced by demographic characteristics, visual abilities, and symptoms experienced. Visual acuity improves a few days after the femtosecond laser–assisted in situ keratomileusis (FS‐LASIK) procedure and 2 weeks after the SMILE procedure and then stabilizes 1–3 months after surgery. Patients often complain of glare, pain, and dry eyes. Recent research has shown that complaints of dry eyes are the main cause of decreased quality of life in patients after refractive surgery. This is caused by a decrease in the thickness of the nerves in the cornea, which patients can feel after surgery, and often improves 3–6 months postsurgery. Postoperative patient quality of life can be assessed objectively using the modulation transfer function, objective scatter index, and aberration examination, whereas subjective assessment uses a questionnaire. Frequently used questionnaires include the Refractive Status Visual Profile, Quality of Life Impact of Refractive Correction, Quality of Vision, Patient Reported Outcome with LASIK, and the National Eye Institute Refractive Error Quality of Life Instrument (NEI‐RQL 42) [11–15].

The NEI‐RQL 42 questionnaire is often used to measure the quality of life and visual function in patients with visual acuity disorders. The questionnaire consists of 42 questions grouped into 13 categories. This questionnaire has been translated into Indonesian and is considered to have high reliability and validity for use in patients with decreased visual acuity. Therefore, this questionnaire was chosen for this study [15–18]. This study aimed to compare the improvement in patient quality of life before and after the SMILE and FS‐LASIK procedures.

## 2. Patients and Methods

This was a nonrandomized, prospective, analytical, and observational study. The study population included all patients who underwent SMILE and FS‐LASIK procedures at the National Eye Center LASIK Center, Cicendo Eye Hospital. The research was conducted between February and May 2024 after obtaining approval from the RSMC PMN Ethics Committee. Informed consent to participate was obtained from all of the participants in the study.

The LASIK procedure begins with the patient lying on the operating table, with a suction ring placed on the eye to fix it. The flap can then be formed using a femtosecond laser. Once the flap is formed, the surgeon opens the flap and turns on the excimer laser to modify the stroma. After the laser treatment is complete, the surgeon returns the flap to its original position. In contrast, the SMILE procedure uses a femtosecond laser to create a lenticular layer that is extracted through a small incision in the cornea without the aid of an excimer laser. In the SMILE procedure, the patient lies on the operating table, and the patient’s eyes are placed on the contact glass of the femtosecond laser. The patient is then asked to look at the green dot, and the suction port is activated to fixate the eye. The lower part of the lenticule is then shaped using an outside‐in method, followed by the upper part using an inside‐out method, and a 2–3 mm opening incision is made in the superotemporal cornea. The incision is then opened, the upper and lower parts of the lenticule are separated, and the lenticule is removed from the cornea using micro‐forceps. The procedure was performed using the VisuMax 500 femtosecond laser and the Visx Star S4 IR excimer laser. The optical zones used in FS‐LASIK were 6.0–6.5 mm and in SMILE were 6.3–6.8 mm. All patients were given artificial tears postoperatively, which may be masking the dry eye condition.

The inclusion criteria were patients who met the requirements to undergo the SMILE or FS‐LASIK procedure, underwent the SMILE or FS‐LASIK procedure on both eyes, were willing to participate, filled out a questionnaire before and after the SMILE or FS‐LASIK procedure, and had never undergone a surgical procedure for eyes. Exclusion criteria were patients who experienced complications during and after the SMILE or FS‐LASIK procedure and those were unable to complete the questionnaire until 1 month after the SMILE or FS‐LASIK procedure.

Research data were analyzed using SPSS Version 24.0 for Windows (IBM Corp., Armonk, NY, USA). Data are presented in the form of percentages (%) for categorical variables and mean ± standard deviation and median for numerical variables. Statistical tests were used to compare the means of numerical variables between the two groups using the unpaired *t*‐test and Mann–Whitney *U* test. Meanwhile, statistical analysis for categorical data were performed using the chi‐square test if the chi‐square requirements were met; if not met, Fisher’s exact test was used for 2 × 2 tables and the Kolmogorov–Smirnov test for tables other than 2 × 2. The chi‐square requirement is that there are no expected values ≤ 5 in 20% of the table. Statistical significance was set at *p* value < 0.05.

## 3. Results

**FIGURE 1:**
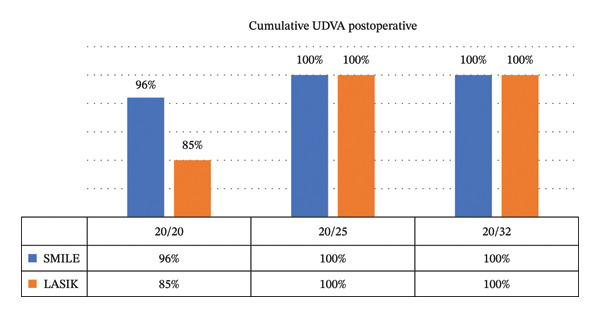
Postoperative cumulative UDVA after SMILE and LASIK.

**FIGURE 2:**
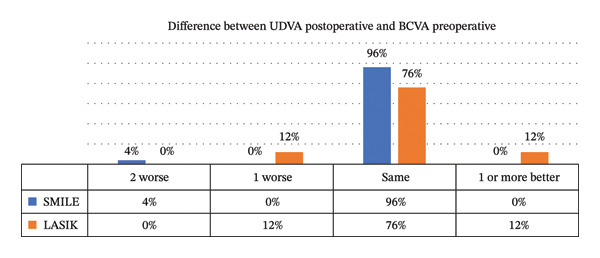
Postoperative UDVA versus preoperative BCVA after SMILE and LASIK.

**FIGURE 3:**
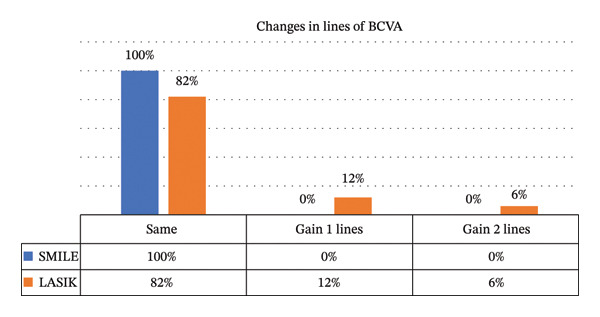
Postoperative changes in BCVA after SMILE and LASIK.

In this study, 59 patients met the inclusion criteria during the study period, 35 patients underwent the FS‐LASIK procedure, and 24 patients underwent the SMILE procedure. Two patients in the FS‐LASIK group dropped out because they could not come for follow‐up and could not be contacted. Figure 1 represents the efficacy of the procedure, which shows that in the SMILE group, 96% had a postoperative UDVA of 20/20 compared to 85% in the LASIK group. Figure 2 shows that 96% in the SMILE group had UDVA preoperative better equal to or better than BCVA preoperative, as compared to 88% in the LASIK group. Figure 3 represents the safety of the procedure, which shows that there are no eyes that lost lines of BCVA in both groups.

Table 1 shows a comparison of the baseline characteristics of the study patients. Numerical data were analyzed using the Mann–Whitney *U* test because the data, including age, SE, and NIBUT, were not normally distributed. Categorical data, including sex and SE category, were analyzed using the chi‐square test. The education variable and NIBU category were analyzed using the Fisher’s exact test, and occupation using the Kolmogorov–Smirnov test. Based on the results of the comparative analysis of the characteristics of the two groups, no differences were observed in the characteristics at the start of the examination. This shows that the two groups were the same or homogeneous, meaning that they were suitable for comparison and further hypothesis testing, except in the SE category.

**TABLE 1:** Comparison of the characteristics of the study patients.

Variable	Group		*p* value
	SMILE	FS‐LASIK	
	*N* = 24	*N* = 33	
Age			0.088
Mean ± Std	20.96 ± 5.229	19.88 ± 4.219	
Median	19	18	
Range (min–max)	17.00–40.00	17.00–36.00	
Gender			0.926
Laki‐laki	17 (70.8%)	23 (69.7%)	
Perempuan	7 (29.2%)	10 (30.3%)	
Education			0.261
Secondary	19 (79.2%)	30 (90.9%)	
Higher	5 (20.8%)	3 (9.1%)	
Occupation			0.991
Students	19 (79.2%)	30 (90.9%)	
Work	5 (20.8%)	2 (6.1%)	
Does not work	0 (0.0%)	1 (3.0%)	
SE			0.808
Mean ± Std	−3.38 ± 1.920	−4.28 ± 3.395	
Median	−2.88	−3.13	
Range (min–max)	−8.25	−10.12	
SE category			0.017^∗^
< −3.00 D	12 (50.0%)	16 (48.5%)	
−3.00 to −6.00 D	10 (41.7%)	5 (15.2%)	
> −6.00 D	2 (8.3%)	12 (36.4%)	
NIBUT			0.523
Mean ± Std	11.28 ± 2.795	11.13 ± 2.832	
Median	10.7	11.7	
Range (min–max)	5.60–15.10	6.10–15.00	
NIBUT category			0.776
≤ 10 detik	10 (41.7%)	15 (45.5%)	
> 10 detik	14 (58.3%)	18 (54.5%)	

*Note:* For numerical data, the *p*‐value was tested using the unpaired t‐test if the data were normally distributed and the alternative Mann–Whitney test if the data were not normally distributed. Categorical data *p*‐values were calculated based on the chi‐square test or the alternative Kolmogorov–Smirnov and Fisher’s exact tests if the chi‐square requirements were not met. *p* values less than 0.05 were considered statistically significant, with ^∗^ indicating *p* < 0.05 and ^∗∗^ indicating *p* < 0.01.

Table 2 shows the comparison of NEI‐RQL 42 questionnaire scores in the SMILE surgery group. The results of the statistical tests in the research group showed that glare was not statistically significant (*p*‐value > 0.05). Meanwhile, other variables were statistically significant (*p*‐value < 0.05).

**TABLE 2:** Comparison of NEI‐RQL 42 questionnaire scores in the SMILE group.

Variable	SMILE		*p* value
	Preoperation	Postoperation	
	*N* = 24	*N* = 24	
Clarity of vision			0.0001^∗∗^
Mean ± Std	42.00 ± 20.218	83.25 ± 18.548	
Median	36.5	92	
Range (min–max)	13.00–100.00	23.00–100.00	
Expectations			0.011^∗^
Mean ± Std	15.63 ± 31.114	38.54 ± 36.845	
Median	0	50	
Range (min–max)	0.00–100.00	0.00–100.00	
Near vision			0.0001^∗∗^
Mean ± Std	66.54 ± 24.999	90.88 ± 14.214	
Median	74	100	
Range (min–max)	19.00–100.00	54.00–100.00	
Far vision			0.0001^∗∗^
Mean ± Std	65.63 ± 25.360	89.08 ± 15.424	
Median	70	97.5	
Range (min–max)	17.00–100.00	43.00–100.00	
Diurnal fluctuations			0.0001^∗∗^
Mean ± Std	49.54 ± 28.733	85.88 ± 21.616	
Median	50	100	
Range (min–max)	0.0–88.00	33.00–100.00	
Activity limitations			0.009^∗^
Mean ± Std	70.25 ± 26.054	87.13 ± 19.896	
Median	75	100	
Range (min–max)	19.00–100.00	38.00–100.00	
Glare			0.486
Mean ± Std	59.04 ± 28.214	64.33 ± 23.766	
Median	75	63	
Range (min–max)	0.00–100.00	25.00–100.00	
Symptoms			0.022^∗^
Mean ± Std	68.75 ± 19.136	77.63 ± 16.352	
Median	69.5	79	
Range (min–max)	36.00–96.00	46.00–100.00	
Dependence on correction			0.0001^∗∗^
Mean ± Std	60.88 ± 31.855	91.33 ± 21.855	
Median	67	100	
Range (min–max)	17.00–100.00	17.00–100.00	
Worry			0.006^∗^
Mean ± Std	25.75 ± 26.019	53.25 ± 35.393	
Median	19	56.5	
Range (min–max)	0.00–88.00	0.00–100.00	
Suboptimal correction			0.019^∗^
Mean ± Std	65.29 ± 33.981	87.04 ± 17.417	
Median	69	100	
Range (min–max)	0.00–100.00	5.00–100.00	
Appearance			0.011^∗^
Mean ± Std	59.96 ± 20.314	77.38 ± 24.306	
Median	53	93	
Range (min–max)	20.00–93.00	13.00–100.00	
Satisfaction with correction			0.019^∗^
Mean ± Std	74.17 ± 20.834	85.83 ± 11.001	
Median	80	80	
Range (min–max)	20.00–100.00	60.00–100.00	
Average			0.0001^∗∗^
Mean ± Std	51.67 ± 11.798	82.29 ± 10.861	
Median	50	75	
Range (min–max)	29.00–74.00	42.00–87.00	

*Note:* For numerical data, the *p*‐value was tested using the paired t‐test if the data were normally distributed, or the Wilcoxon test if the data were not normally distributed. *p* values less than 0.05 were considered statistically significant, with ^∗^ indicating *p* < 0.05 and ^∗∗^ indicating *p* < 0.01.

Table 3 shows the comparison of NEI‐RQL 42 questionnaire scores in the LASIK surgery group. The results of the statistical tests in the research group showed that activity limitation, glare, symptoms, worry, suboptimal correction, and appearance were not statistically significant (*p*‐value > 0.05). Meanwhile, clarity of vision, patient expectations, near vision, far vision, diurnal fluctuations, dependence on correction, satisfaction with correction, and average questionnaire value were statistically significant (*p*‐value < 0.05).

**TABLE 3:** Comparison of NEI‐RQL 42 questionnaire scores in the FS‐LASIK group.

Variable	FS‐LASIK		*p* value
	Preoperation	Postoperation	
	*N* = 33	*N* = 33	
Clarity of vision			0.0001^∗∗^
Mean ± Std	49.73 ± 24.227	77.33 ± 16.110	
Median	50	81	
Range (min–max)	0.00–100.00	40.00–100.00	
Expectations			0.001^∗^
Mean ± Std	15.91 ± 22.377	38.64 ± 41.969	
Median	0	50	
Range (min–max)	0.00–50.00	0.00–100.00	
Near vision			0.0001^∗∗^
Mean ± Std	69.82 ± 21.947	89.67 ± 10.325	
Median	73	92	
Range (min–max)	13.00–100.00	67.00–100.00	
Far vision			0.0001^∗∗^
Mean ± Std	65.85 ± 25.785	86.06 ± 12.850	
Median	73	88	
Range (min–max)	0.00–100.00	50.00–100.00	
Diurnal fluctuations			0.0001^∗∗^
Mean ± Std	58.12 ± 21.817	81.88 ± 16.013	
Median	58	88	
Range (min–max)	13.00–100.00	46.00–100.00	
Activity limitations			0.367
Mean ± Std	72.15 ± 27.360	76.73 ± 27.340	
Median	75	88	
Range (min–max)	0.00–100.00	0.00–100.00	
Glare			0.426
Mean ± Std	53.27 ± 24.399	49.94 ± 17.207	
Median	50	50	
Range (min–max)	0.00–100.00	13.00–100.00	
Symptoms			0.135
Mean ± Std	73.79 ± 19.046	68.64 ± 16.483	
Median	75	71	
Range (min–max)	32.00–100.00	39.00–100.00	
Dependence on correction			0.0001^∗∗^
Mean ± Std	54.06 ± 32.287	91.18 ± 12.854	
Median	58	100	
Range (min–max)	0.00–100.00	58.00–100.00	
Worry			0.127
Mean ± Std	27.15 ± 21.338	36.48 ± 25.510	
Median	25	38	
Range (min–max)	0.00–75.00	0.00–100.00	
Suboptimal correction			0.491
Mean ± Std	77.00 ± 25.966	81.12 ± 26.481	
Median	88	100	
Range (min–max)	25.00–100.00	13.00–100.00	
Appearance			0.09
Mean ± Std	57.58 ± 27.147	67.55 ± 28.277	
Median	53	87	
Range (min–max)	0.00–100.00	7.00–100.00	
Satisfaction with correction			0.049^∗^
Mean ± Std	69.70 ± 20.076	78.18 ± 18.950	
Median	60	80	
Range (min–max)	20.00–100.00	20.00–100.00	
Average			0.0001^∗∗^
Mean ± Std	53.12 ± 12.341	66.00 ± 7.710	
Median	55	67	
Range (min–max)	25.00–77.00	50.00–79.00	

*Note:* For numerical data, the *p*‐value was tested using the paired t‐test if the data were normally distributed, or the Wilcoxon test if the data were not normally distributed. *p* values less than 0.05 were considered statistically significant, with ^∗^ indicating *p* < 0.05 and ^∗∗^ indicating *p* < 0.01.

Table 4 shows the comparison of the improvement in NEI‐RQL 42 questionnaire scores between the FS‐LASIK and SMILE surgery groups. The results of the statistical tests in the research group showed that delta clarity of vision, delta patient expectations, delta near vision, delta far vision, delta diurnal fluctuations, delta activity limitation, delta glare, delta dependence on correction, delta worry, delta correction suboptimal, delta appearance, and delta satisfaction regarding correction were not statistically significant (*p*‐value > 0.05). Meanwhile, delta symptoms and delta average questionnaire value were statistically significant (*p*‐value < 0.05).

**TABLE 4:** Comparison of the improvement in NEI‐RQL 42 questionnaire scores in the SMILE and FS‐LASIK groups one month postsurgery.

Variable	Group		*p* value
	SMILE	FS‐LASIK	
	*N* = 24	*N* = 33	
Delta clarity of vision			0.067
Mean ± Std	41.25 ± 26.822	27.55 ± 27.634	
Median	44	31	
Range (min–max)	−8–83	−29–92	
Delta expectations			0.884
Mean ± Std	22.92 ± 36.053	22.73 ± 32.091	
Median	25	0	
Range (min–max)	−50–100	−50–100	
Delta near vision			0.496
Mean ± Std	24.42 ± 24.596	19.91 ± 24.471	
Median	16	13	
Range (min–max)	−8–69	−27–81	
Delta far vision			0.315
Mean ± Std	23.54 ± 19.576	20.21 ± 28.308	
Median	19	10	
Range (min–max)	0–77	−42–89	
Delta diurnal fluctuations			0.118
Mean ± Std	36.33 ± 32.047	23.67 ± 27.963	
Median	37.5	17	
Range (min–max)	−29–100	−29–83	
Delta activity limitations			0.087
Mean ± Std	17.04 ± 26.756	4.70 ± 25.064	
Median	16	0	
Range (min–max)	−31–69	−38–63	
Delta glare			0.262
Mean ± Std	5.33 ± 30.625	−3.42 ± 27.470	
Median	13	0	
Range (min–max)	−50–75	−50–63	
Delta symptoms			0.006^∗^
Mean ± Std	9.00 ± 17.935	−5.21 ± 19.207	
Median	4	−4	
Range (min–max)	−25–46	−43–46	
Delta dependence on correction			0.422
Mean ± Std	30.54 ± 32.378	37.21 ± 35.662	
Median	23	33	
Range (min–max)	−17–83	−29–100	
Delta worry			0.067
Mean ± Std	27.67 ± 42.131	9.52 ± 31.267	
Median	19	0	
Range (min–max)	−88–100	−63–75	
Delta suboptimal correction			0.125
Mean ± Std	21.92 ± 39.686	4.15 ± 28.676	
Median	19	0	
Range (min–max)	−38–100	−50–75	
Delta appearance			0.415
Mean ± Std	17.42 ± 30.116	10.15 ± 34.897	
Median	13	7	
Range (min–max)	−40–73	−73–80	
Delta satisfaction with correction			0.559
Mean ± Std	11.67 ± 21.196	8.48 ± 22.929	
Median	10	0	
Range (min–max)	−40–60	−40–60	
Delta average			0.036^∗^
Mean ± Std	20.63 ± 14.206	12.79 ± 13.105	
Median	22.5	13	
Range (min–max)	−2–50	−16–53	

*Note:* For numerical data, the *p*‐value was tested using the unpaired t‐test if the data were normally distributed, or the Mann–Whitney test if the data were not normally distributed. *p* values less than 0.05 were considered statistically significant, with ^∗^ indicating *p* < 0.05 and ^∗∗^ indicating *p* < 0.01.

Table 5 shows the comparison of the increase in NEI‐RQL 42 questionnaire scores in the SE group. The results of the statistical tests showed that delta clarity of vision, delta patient expectations, delta near vision, delta diurnal fluctuations, delta activity limitations, delta glare, delta symptoms, delta worry, delta appearance, delta satisfaction with over correction were not statistically significant (*p*‐value > 0.05). Meanwhile, delta far vision, delta dependence on correction, delta suboptimal correction, and delta average questionnaire were statistically significant (*p*‐value < 0.05).

**TABLE 5:** Comparison of the increase in NEI‐RQL 42 questionnaire scores in the SE group.

Variable	SE			*p* value
	<−3.00	−3.00 to −6.00	>−6.00	
	*N* = 28	*N* = 15	*N* = 14	
Delta clarity of vision				0.649
Mean ± Std	30.39 ± 27.275	33.47 ± 33.387	39.00 ± 23.547	
Median	34	33	35.5	
Range (min–max)	−21–92	−29–71	−13–69	
Delta expectations				0.31
Mean ± Std	17.86 ± 33.923	35.00 ± 33.806	19.64 ± 31.284	
Median	0	50	12.5	
Range (min–max)	−50–100	0.00–100.00	−50–50	
Delta near vision				0.054
Mean ± Std	12.54 ± 13.273	27.93 ± 31.072	33.79 ± 27.882	
Median	13	35	41	
Range (min–max)	−8–46	−27–69	−8–81	
Delta far vision				0.020^∗^
Mean ± Std	11.68 ± 11.166	25.73 ± 27.120	37.07 ± 33.495	
Median	10	25	44.5	
Range (min–max)	0–40	−42–77	−7–89	
Delta diurnal fluctuations				0.075
Mean ± Std	20.21 ± 22.806	33.87 ± 40.060	41.36 ± 27.326	
Median	17	29	48	
Range (min–max)	−29–58	−29–100	−21–83	
Delta activity limitations				0.094
Mean ± Std	1.86 ± 22.105	18.73 ± 27.696	16.50 ± 29.301	
Median	0	19	7.5	
Range (min–max)	−38–69	−38–63	−38–63	
Delta glare				0.969
Mean ± Std	0.04 ± 31.757	−0.80 ± 20.592	1.86 ± 32.304	
Median	0	0	13	
Range (min–max)	−50–75	−38–38	−50–63	
Delta symptoms				0.206
Mean ± Std	5.54 ± 17.815	−3.53 ± 21.751	−4.14 ± 20.691	
Median	2	−4	−7.5	
Range (min–max)	−25–46	−43–36	−29–46	
Delta dependence on correction				0.0001^∗∗^
Mean ± Std	14.46 ± 19.163	39.73 ± 35.660	68.57 ± 27.467	
Median	13	42	83	
Range (min–max)	−21–29	−29–83	0–100	
Delta worry				0.278
Mean ± Std	10.29 ± 38.621	29.33 ± 35.658	17.86 ± 34.038	
Median	6.5	25	12.5	
Range (min–max)	−88–100	−38–100	−38–75	
Delta suboptimal correction				0.041^∗^
Mean ± Std	2.21 ± 29.578	32.53 ± 37.517	8.07 ± 33.262	
Median	0	38	6.5	
Range (min–max)	−50–100	−25–100	−38–75	
Delta appearance				0.233
Mean ± Std	20.04 ± 29.535	11.07 ± 33.223	1.86 ± 37.601	
Median	10	7	0	
Range (min–max)	−40–80	−73–53	−67–73	
Delta satisfaction with correction				0.565
Mean ± Std	12.86 ± 17.397	5.33 ± 25.598	8.57 ± 26.849	
Median	10	0	20	
Range (min–max)	−20–60	−40–60	−40–60	
Delta average				0.042^∗^
Mean ± Std	11.39 ± 9.299	20.53 ± 18.051	20.71 ± 14.886	
Median	12.5	24	21.5	
Range (min–max)	−10–30	−16–50	−5–53	

*Note:* For numerical data, the *p*‐value was tested using the one‐way ANOVA test if the data were normally distributed, or the Kruskal–Wallis test if the data were not normally distributed. *p* values less than 0.05 were considered statistically significant, with ^∗^ indicating *p* < 0.05 and ^∗∗^ indicating *p* < 0.01.

## 4. Discussion

Refractive errors are the main causes of visual impairment that can be corrected. Refractive disorders such as astigmatism and myopia are often found in young adults with an average age of approximately 20 years. This is in accordance with the research group in this study, whose average age was approximately 20 years. Idan et al. showed that surgery in older adult patients has a lower success rate than that in young adult patients, whereas adolescent patients have the same success rate as young adult patients [2, 19–21].

The patients who underwent refractive surgery in this study were predominantly men and students with secondary education levels. This is related to the goal of most patients, namely, to enroll in a military school that requires applicants to be free from refractive aids such as glasses and contact lenses. The aim is to ensure that the participants do not depend on assistive devices in war and to prevent infections caused by wearing contact lenses in the field. Research conducted by Godiwalla et al. showed that postrefractive surgery patients who worked in the military had clear and stable vision and could perform their duties well [4, 20, 22].

In this study, the average refractive error was −3.38 D and −4.28 D in the SMILE and FS‐LASIK groups, respectively. This is in accordance with a study by Uchakan et al., who stated that the average refractive error of patients undergoing refractive surgery was −2.50 D to −5.00 D. Patients with severe myopia undergo this procedure more frequently than those with mild myopia. There were significant differences in refractive error categories in the study population, with the FS‐LASIK group having more patients with more severe refractive errors [23–26].

The success rate of refractive surgery is often determined by its efficacy, stability, predictability, and safety. Although these parameters are important, they should be accompanied by the patient’s subjective report to assess the overall success of the surgery. SMILE and FS‐LASIK are the most frequently performed refractive surgical procedures. The SMILE technique involves a smaller corneal incision, thereby reducing damage to the cornea and preserving its structure and function. In this study, although good visual acuity was obtained after surgery in both groups, there were differences in the quality of life scores between the two groups [7, 27, 28].

Pre‐ and postoperative comparisons in this study showed that in the SMILE group, there was an increase in the postoperative questionnaire scores for all variables, except glare. In the FS‐LASIK group, there was an increase in the postoperative questionnaire scores for almost all variables, except for glare and symptom variables, which showed a decrease in value but were not statistically significant. This is in accordance with previous research, which stated that there was a significant increase in quality of life after refractive surgery. In addition, Rose et al. assessed the quality of life using the NEI‐RQL 42 questionnaire in military training patients after undergoing refractive surgery and obtained significant improvements in the variable clarity of vision, patient expectations, near vision, distance vision, diurnal fluctuations, activity limitations, glare, and reliance on correction. There were no significant differences in symptom variables [11, 13, 27–29].

Improvements in visual clarity, patient expectations, activity limitations, concerns, appearance, and satisfaction with corrections are often associated with postoperative visual acuity recovery. Several studies have reported that FS‐LASIK surgery has a faster rate of recovery of postoperative visual acuity, resulting in higher questionnaire scores compared to patients after SMILE surgery. In this study, post‐SMILE surgery patients had a higher increase in these variables, but this was not statistically significant, which could also be influenced by the distribution of the degree of myopia, which was more severe in the FS‐LASIK group [27, 30, 31].

Shengyu et al. compared the refractive surgical procedures for high myopia. This study showed that the SMILE procedure produces a larger functional optical zone and a thicker cornea than the FS‐LASIK procedure. This may affect postoperative symptoms, such as glare and halos. In this study, glare symptoms were more common in the FS‐LASIK group, but the difference was not statistically significant [23, 27, 29].

In this study, a comparison of the improvement in quality of life scores between SMILE and FS‐LASIK patients showed higher scores in the SMILE group for all variables, except for dependence on correction. This increase was considered statistically significant for symptom variables and average questionnaire scores. The FS‐LASIK procedure involves creating a flap that causes corneal nerve damage and increases the risk of postoperative dry eye symptoms. Yingjie et al. showed that post‐SMILE surgery patients had higher TBUT and minimal dry eye symptoms than post‐FS‐LASIK surgery patients. The difference in symptom variable values resulted in a significant difference in the mean questionnaire scores between the SMILE and FS‐LASIK procedures, indicating that the improvement in quality of life scores in the SMILE group was the result of early symptom relief. This is in accordance with previous studies reporting that the quality of life of patients after SMILE surgery was better than that of patients after FS‐LASIK surgery [27, 31–33].

In this study, there was a greater increase in questionnaire scores in the SMILE group in relation to the dependence on correction variable. This is because there were differences in the SE group, which was more severe after FS‐LASIK surgery. Analysis of the increase in quality of life scores based on SE group showed that patients in the more severe SE group had a significantly higher increase in quality of life scores, especially in the dependence on correction variable. This led to a greater increase in questionnaire scores in the FS‐LASIK group. Research by Yanyan et al. did not show any difference in the success and quality of life after SMILE and FS‐LASIK surgery in patients with severe myopia; therefore, the difference in increasing this value is likely caused by differences in the SE group [23–26].

A limitation of this study was that 18 patients could not come for follow‐up due to cost and distance; therefore, these patients could not have the questionnaire examined directly. In addition, no matching was carried out in the study population because it is set in private hospitals; therefore, it is prone to selection bias. Several studies also stated the significance of refractive error degree in patients’ quality of life post refractive procedure; as there are significant differences in study populations between SMILE and FS‐LASIK, the results of this study must be taken carefully. This study only compared the quality of life 1 month pre‐ and postoperatively, while dry eye complaints and postoperative symptoms usually improve after 6 months. All postoperative patients were administered artificial tear drops so that dry eye symptoms could be disguised and affect the improvement in the patient’s quality of life.

## 5. Conclusions

In conclusion, in this study, there was a greater increase in quality of life scores in patients after the SMILE procedure than after the FS‐LASIK procedure. This research can serve as a reference for providing informed consent to patients who will undergo refractive surgery regarding postoperative quality of life, thus helping them determine the desired surgical procedure. In future research, it will be possible to assess changes in NIBUT pre‐ and postrefractive surgery and relate them to patient complaints using a questionnaire to objectively and subjectively compare postoperative dry eye complaints of patients.

**Nomenclature**
LASIK: Laser‐assisted in situ keratomileusis surgery
FS‐LASIK: Femtosecond laser‐assisted in situ keratomileusis
SMILE: Small incision lenticule extraction
RSVP: Refractive status visual profile
QIRC: Quality of life impact of refractive correction
QoV: Quality of vision
PROWL: Patient reported outcome with LASIK
NEI‐RQL42: National Eye Institute Refractive Error Quality of Life Instrument
WHO: World Health Organization
IAPB: International Agency for Prevention of Blindness
PRK: Photorefractive keratectomy
TBUT: Tear break‐up time
NIBUT: Non‐invasive break‐up time
UDVA: Uncorrected distance visual acuity
BCVA: Best corrected visual acuity
SE: Spherical equivalent
PMN: Pusat Mata Nasional
RSMC: Rumah Sakit Mata Cicendo

## Author Contributions

Pieter Juanarta contributes to the conception, design, analysis, and interpretation of data. Susanti Natalya Sirait contributes to design, analysis, and interpretation of data. R. Angga Kartiwa contributes to conception, analysis, and interpretation of data. Feti Karfiati contributes to design, analysis, and interpretation of data. Rova Virgana contributes to analysis, and interpretation of data. Budiman contributes to the conception, acquisition, and analysis of data.

## Funding

This research was funded by National Eye Center Cicendo Eye Hospital Bandung.

## Disclosure

All authors have approved the submitted version and agreed both to be personally accountable for the author’s own contributions and to ensure that questions related to the accuracy or integrity of any part of the work, even ones in which the author was not personally involved, are appropriately investigated, resolved, and the resolution documented in the literature.

## Ethics Statement

The research was conducted at the LASIK Center polyclinic and Aesthetic Eye Care & Dry Eye Clinic, National Eye Center Cicendo Eye Hospital, Bandung, from February to May 2024. This research has received approval from the PMN Ethics Committee of Cicendo Eye Hospital with Number: DP.04.03/D.XXIV.16/2745/2024. Informed consent to participate was obtained from all of the participants in the study.

## Conflicts of Interest

The authors declare no conflicts of interest.

## Supporting Information

Additional supporting information can be found online in the Supporting Information section.

## Supporting information


**Supporting Information** The data that has been collected and used in this research is available in the supplementary material section.

## Data Availability

The data that support the findings of this study are available in the Supporting Information of this article.
